# Ernst Rüdin’s Unpublished 1922-1925 Study “Inheritance of Manic-Depressive Insanity”: Genetic Research Findings Subordinated to Eugenic Ideology

**DOI:** 10.1371/journal.pgen.1005524

**Published:** 2015-11-06

**Authors:** Gundula Kösters, Holger Steinberg, Kenneth Clifford Kirkby, Hubertus Himmerich

**Affiliations:** 1 Archives for the History of Psychiatry in Leipzig, Department of Psychiatry and Psychotherapy, University of Leipzig, Leipzig, Germany; 2 Claussen-Simon-Endowed Professorship for Neurobiology of Affective Disorders, Department of Psychiatry and Psychotherapy, University of Leipzig, Leipzig, Germany; 3 Department of Psychiatry, University of Tasmania, Hobart, Tasmania, Australia; Seattle Children's Research Institute, UNITED STATES

## Abstract

In the early 20^th^ century, there were few therapeutic options for mental illness and asylum numbers were rising. This pessimistic outlook favoured the rise of the eugenics movement. Heredity was assumed to be the principal cause of mental illness. Politicians, scientists and clinicians in North America and Europe called for compulsory sterilisation of the mentally ill. Psychiatric genetic research aimed to prove a Mendelian mode of inheritance as a scientific justification for these measures. Ernst Rüdin’s seminal 1916 epidemiological study on inheritance of dementia praecox featured large, systematically ascertained samples and statistical analyses. Rüdin’s 1922–1925 study on the inheritance of “manic-depressive insanity” was completed in manuscript form, but never published. It failed to prove a pattern of Mendelian inheritance, counter to the tenets of eugenics of which Rüdin was a prominent proponent. It appears he withheld the study from publication, unable to reconcile this contradiction, thus subordinating his carefully derived scientific findings to his ideological preoccupations. Instead, Rüdin continued to promote prevention of assumed hereditary mental illnesses by prohibition of marriage or sterilisation and was influential in the introduction by the National Socialist regime of the 1933 “Law for the Prevention of Hereditarily Diseased Offspring” (*Gesetz zur Verhütung erbkranken Nachwuchses*).

## Author Summary

Psychiatric genetics was established as a scientific discipline in the early 20^th^ century. The intention was to provide evidence for the inheritance of mental illnesses. This was to open up new paths in prevention, since therapeutic options at the time were meagre. Applying modern study designs and statistical methods, the German psychiatrist Ernst Rüdin (1874–1952) found lower inheritance of affective disorders than anticipated. He surmised that external factors were also important in their development. From the vantage point of the present, his results were sound. However, this major study, titled “On the Inheritance of Manic-Depressive Insanity”, though developed as a manuscript, was never published. The question arises whether Rüdin doubted his own scientific methods and hence results, or if he was influenced by his ideological views and the expectations of the scientific community and German society. Whilst withholding his results, he continued to promote prevention of assumed hereditary mental illnesses by prohibition of marriage or sterilisation.

## Introduction

Formal genetic psychiatry was established in the early 20^th^ century at a time of therapeutic nihilism, when psychiatry was strongly influenced by the theory of hereditary degeneration [1–7]. This combined with economic considerations, led to a change of emphasis from healing to prevention of the more severe types of psychiatric illness [1, 3, 8]. This was reflected in the popularity of the eugenics movement, which advocated measures such as restrictions on marriage or sterilisation to prevent inherited disease [3, 9]. A key aim of psychiatric genetic research was to provide scientific evidence that severe mental illnesses were inherited, thus strengthening the case for eugenic measures.

Ernst Rüdin (1874–1952) [11] has been credited as the originator of modern psychiatric human genetics on the basis of his research aiming to establish inheritance estimates, that is the risk of a relative developing the same disease as the index patient, which he termed the *“empirische Erbprognose”* (“empirical heredity prognosis”) [8, 10].

During his formative school and university years, Rüdin’s worldview was heavily influenced by his brother-in-law, the eugenicist Alfred Ploetz (1860–1940), and by Auguste Forel’s (1848–1931) abstinence movement [8] (for detailed biographical information see Table 1).

**Table 1:** Biography of Ernst Rüdin.

19 Apr 1874	Born in St. Gallen, Switzerland [8]
1893–1898	Study of human medicine in Geneva, Heidelberg, Berlin und Zürich, Swiss state examinations [8]
1899–1905	Psychiatric training in Zürich, Berlin, Basel und Heidelberg (Kraepelin) [8]
1901	Doctoral thesis in Zürich: “On Clinical Forms of Prison Psychoses” [12]
1905	Editor of the “Archives of Racial and Social Biology” founded by Ploetz [13, 14]
1907	Director of the Society for Racial Hygiene [14], scientific assistant to Kraepelin at the Psychiatric University Clinic of Munich
1908	Licence to practice medicine for the German Reich, Co-Publisher of the “Archives of Racial and Social Biology” [13]
1909	Senior physician at the Psychiatric University Clinic of Munich, habilitation on psychiatric illnesses of prison inmates [15]
1912	Designation as a Bavarian civil servant, thereby acquiring German citizenship [8]
1918	Head of the Genealogic-Demographic Department (GDA), the world’s first research facility for psychiatric genetics and epidemiology, at the German Research Institute of Psychiatry (*Deutsche Forschungsanstalt für Psychiatrie*, *DFA*) in Munich, founded by Kraepelin in 1917 [13, 16]
1925–1928	Chair of Psychiatry, University of Basel [8]
1928	Honorary professor of the medical faculty of the University of Munich [8]
1931–1945	Managing director of the DFA (since 1924 the Institute of the Kaiser-Wilhelm Society, now the Max Planck Society) [8]
1933	Chairman of the “Committee II for Racial Hygiene and Racial Policy of the Council of Experts of Racial and Population Policy for the Reich Minister of the Interior” *(“Arbeitsgemeinschaft II für Rassenhygiene und Rassenpolitik des Sachverständigenbeirats für Rassen- und Bevölkerungspolitik”*) [8], collaboration on the “Law for the Prevention of Hereditarily Diseased Offspring” [17]
1933–1939	Initiation in various international professional societies, e.g. in Japan, France and Hungary, and numerous distinctions [8]
1935–1945	Chairman of the Society of German Neurologists and Psychiatrists (*Gesellschaft Deutscher Neurologen und Psychiater*, *GDNP*) [8]
1937	Member of the Nazi Party (NSDAP) [14]
1939–1944	Publisher of the “Archives of Racial and Social Biology”, succeeding Ploetz [8]
1945	Revocation of Swiss citizenship, removal from office by the American military government in Bavaria [18]
1949	Denazification process in Munich: categorised as “*minderbelastet*” (marginally incriminated, Group III) and in 1950 as a “*Mitläufer*” (follower or nominal member, Group IV) [8]
22 Oct 1952	Death in Munich [14]

Rüdin advocated the goals of eugenics early in his career and called, in numerous publications and reviews, for prohibition of marriage and sterilisation of “inferior persons” such as mentally ill and handicapped persons, criminals, alcoholics and prostitutes [15, 19–21].

Rüdin formulated the primary goal of his “psychiatric-genealogical” research, namely to create a scientific basis for racial hygiene measures, thereby solving the “social issue” (*soziale Frage*, social ills of industrialised societies in 19^th^ century Europe [22]). Rüdin, who had worked under Emil Kraepelin (1856–1926) in Heidelberg and Munich, adopted his psychiatric nosology and was influenced by Kraepelin’s belief in a strong hereditary component [8, 15, 23, 24].

Rüdin’s research methodology was based on systematic family studies with large sample sizes, using statistical analyses such as Wilhelm Weinberg’s (1862–1937) age correction, simple sib and proband method [25], to identify Mendelian rules of inheritance, and to generate estimates of genetic risk on this basis [26]. Rüdin applied this methodology for the first time at The German Research Institute of Psychiatry (*Deutsche Forschungsanstalt für Psychiatrie*, *DFA*) in Munich in his 1916 study “On the Problem of the Inheritance and Onset of Dementia Praecox”, which was the foundation of his reputation as a psychiatric geneticist [24, 27]. 701 families of patients with “dementia praecox” (a group of endogenous psychoses [4, 23]) were examined in the study. From his results, Rüdin derived the hypothesis of a two-locus recessive model, though he did not obtain conclusive proof of recessive inheritance.

### Rüdin’s study “Inheritance of manic-depressive insanity”

At the DFA, Rüdin then worked on his second major inheritance study, on the genetic inheritance of “manic-depressive insanity” (*Zur Vererbung des manisch-depressiven Irreseins*) [28]. The original manuscript in the Historical Archives of the Max Planck Institute of Psychiatry in Munich is undated; on the basis of the publications cited the compilation can be dated to the period 1922–1925 [8]. It was never published despite being intended for chapter 4 of “Studies on the Inheritance and Origin of Mental Illness”, one of a series of monographs from the publisher Springer-Verlag of Berlin, covering the full domain of neurology and psychiatry [29]. The Rüdin biographer and psychiatrist Matthias Weber described the study as “the most comprehensive and probably most significant of Rüdin’s works” [8].

The manuscript consists of approximately 160 pages of unbound typescript, each chapter paginated separately, with Rüdin’s hand-written corrections, and numerous large-format, hand-written charts [28]. Some chapters are incomplete and untitled and are not considered in this paper. An additional 250 pages of Rüdin’s hand-written notes, unpaginated, unsystematised and partially in shorthand are mostly illegible. The bibliography is hand-written and only partially intelligible.

Rüdin characterised the study as a complement to his 1916 dementia praecox study and used the same methodology [24]. Whilst not excluding a role for environmental factors, he assumed inheritance of a disposition to affective disorders as generally acknowledged but unproven, citing Kraepelin [30]. Rüdin predicted Mendelian dominant inheritance of affective disorders [26]. He intended to prove this on the basis of the segregation pattern in the families of his probands, or determine another mode of Mendelian inheritance. Criteria could then be derived for selection of persons for the eugenic measures he had formulated earlier [20].

The inclusion criteria encompassed all patients admitted as inpatients to the Psychiatric University Clinic of Munich (*Psychiatrische Universitätsklinik München*) with a diagnosis of “manic-depressive insanity” since 1904. No preference was given to patients with a dense family history; the diagnosis alone was the criterion for inclusion. This type of systematic ascertainment was unusual in psychiatry at a time when spectacular single case studies or densely affected families were the main focus of research [31, 32]. Follow-ups were conducted to evaluate the course of the illness and verify the diagnosis.

Rüdin sought accurate clinical diagnostics for inclusion and exclusion of probands, using Kraepelinian nosology, which was widely recognised though not without controversy [33, 34]. Under the diagnostic rubric of “manic-depressive insanity”, Kraepelin included “periodic and circular insanity”, “simple mania”, “melancholy” and “amentia” [30]. This corresponds approximately to the modern-day categorisation of affective disorders [35–38].

For the diagnosis Rüdin depended above all on the clinical symptoms and less on the course of the disease. Kraepelin regarded “manic-depressive insanity” as “curable” and dementia praecox as “incurable”. Rüdin emphasised however that cases of both disorders were documented that did not follow this rule. He called upon a second clinician with no knowledge of the family history to verify the diagnoses [28]. The sample were hospitalised patients, thus biasing to more severe cases [15]. The patients and, whenever possible, family members were interviewed; further sources included medical and administrative files. However, when a medical diagnostic interview could not be arranged for family members, these diagnoses were often based on descriptions of maladjusted or otherwise conspicuous family members, how often this occurred is not recorded. The most important information (age, diagnosis, medical history and family history) was noted on standard cards, as implemented by Kraepelin, and in detailed family trees [24].

Rüdin integrated Weinberg’s statistical methods and Mendelian genetics into a research methodology which sought to predict the passing on of mental illnesses within families: the “empirical heredity prognosis”. His second goal was to prove a Mendelian mode of inheritance via the systematic ascertainment of as many patients with affective disorders as possible. Rüdin recognised the problem that in recessive modes of inheritance, only families in which the disposition was expressed could be counted, while families with heterozygote carriers were lost. In order to be able to apply Mendelian rules to a sample, the ascertained morbidities would also ideally have to correspond to those in the population. Rüdin applied Weinberg’s simple sib and proband method to that end [25]. He stayed in close contact with Weinberg during the evaluation of his results; their correspondence is cited in several places in the manuscript.

The overrepresentation of patients with affective disorders in the sample was corrected by the simple sib method, whereby index cases, referred to as “probands” (*Probanden*), were excluded from the calculation. Affected siblings were entered into the calculation as “secondary cases” (*Sekundärfälle*). The proband method was used to correct for multiple ascertainment in families with several probands.

Rüdin also applied two different methods for age correction, allowing for the possibility that a family member who was healthy at interview could become ill later [28, 39]. On the basis of age distribution, Rüdin determined the period of risk for the onset of affective disorders to be between the ages of 14 and 68. The number of “lifetimes at risk” (*Bezugsziffer*) was then calculated from the sum of siblings, corrected for their age (Table 2).

**Table 2:** Calculation of the morbid risk using the example of category “both parents unaffected” (category I), with the proband method, simple sib method and Weinberg’s abridged age correction.

Calculation of the morbid risk	MR = (N/B)x100 = [N/(0xP1+0,5xP2+1xP3)]x100
	Lifetimes at risk in category “both parents unaffected” with the abridged Weinberg method:
	B = 0xP1+0,5xP2+1xP3
	= 0x1222+0,5x1917+1x118
	= 1076,5
	Affected persons, with the Weinberg simple sibship and proband method:N = 58 secondary cases + 22 probands = 80
Morbid risk for affective disorders in category I	MR = (N/B)x100 = (80/1076,5)x100 = 7,43%
Morbid risk for “other psychiatric conditions” in category I	MR = (N/B)x100 = (101/1076,5)x100 = 9,38%

MR = morbid risk, N = number of affected persons, B = lifetimes at risk (*“Bezugsziffer”*), P1 = number of children aged under 14, P2 = number of persons aged 14–68, P3 = number of persons aged above 68.

Rüdin’s “empirical heredity prognosis” was based on the calculation of a predictive risk of illness, the “morbid risk” (*Morbiditätsrisiko*), which included the above-mentioned Weinberg methods. The morbid risk was calculated from the number of affected persons in the sample, corrected by the simple sib and proband methods, divided by the number of lifetimes at risk, that were calculated by the age correction method (Table 2).

Calculating the morbid risk, Rüdin assumed full penetrance for the inherited traits in question and excluded a possible influence of external factors or interference with other genes. The thus calculated morbid risk was compared with the proportions expected from a Mendelian crossing in order to prove Mendelian inheritance and thereby the inheritance of affective disorders as such. Rüdin also used morbid risk as a predictive value for any particular person with certain preconditions (e.g. a parent with an affective disorder) to develop an affective disorder at some point in their life, and therefore serve as a kind of “genetic counseling”, or as he called it, an “empirical heredity prognosis” [28].

Rüdin’s use of statistical processes was a groundbreaking approach at the beginning of the 20^th^ century and led to sound results that in part remain valid today [32, 40]. However, while focussing on the methods of Weinberg, who was also chairman of the “Society for Racial Hygiene” and, like Rüdin, a staff member of the “Archive of Racial and Social Biology”, Rüdin ignored some other statistical methods, such as correlational analyses, which were in common use [15].

**Fig 1:**
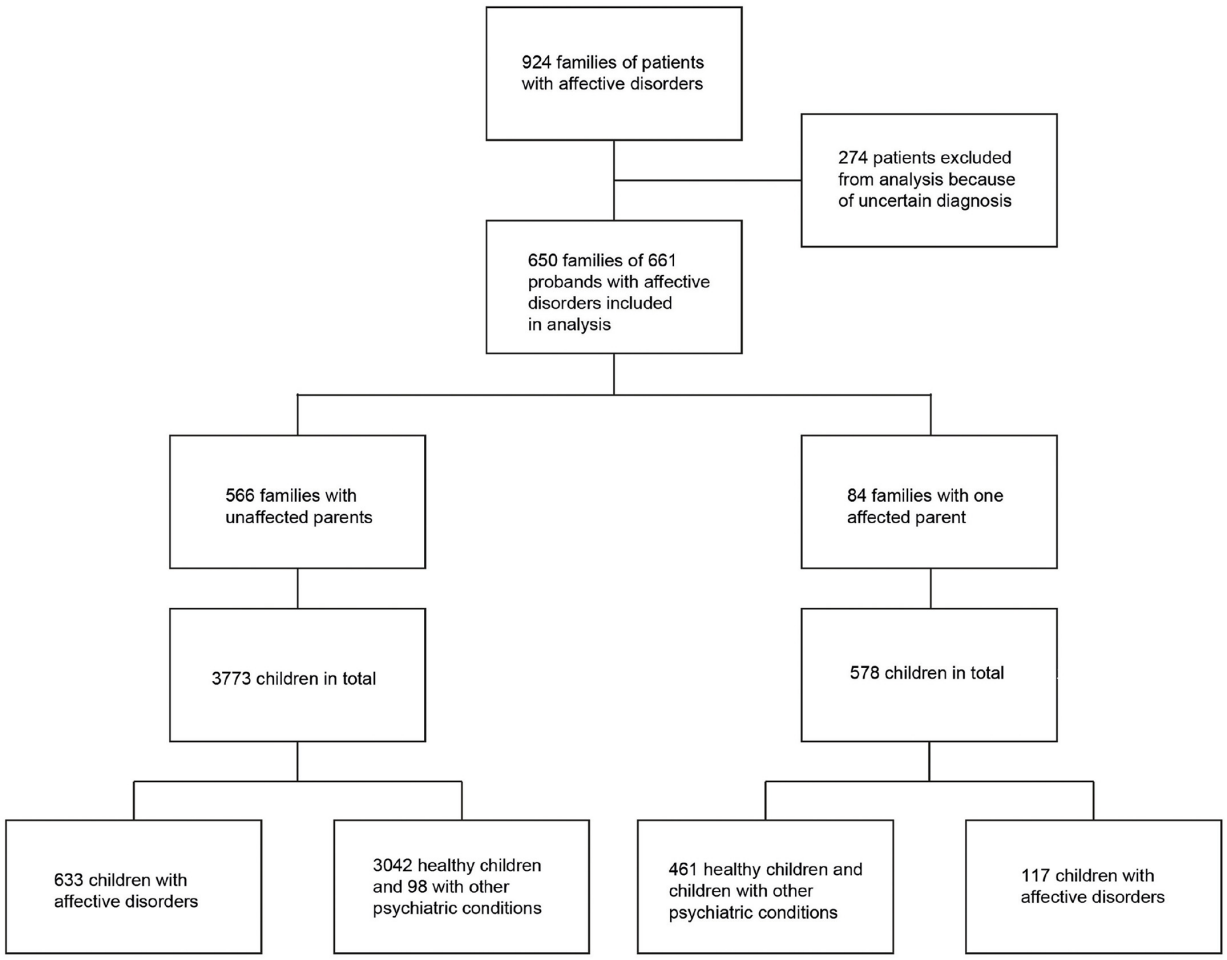
Patients included in the study [28]. The figure was created by the authors on the basis of the data obtained from Rüdin’s manuscript.

After excluding unconfirmed diagnoses, the sample comprised 661 probands from 650 families, or “sibships”, comprised of 4351 siblings in total (Fig 1). In 566 families both parents were healthy and in 84 families one parent had an affective disorder (Table 3).

**Table 3:** Distribution of affective disorders among the siblings of included families.

	Families in total	Affective disorders in the siblings, N (%)	Average number of children per family	Siblings in total
**Both parents unaffected**	566	633 (16.77)	6.67	3773
**One parent affected**	84	117 (20.24)	6.88	578
**Sum**	650	750 (17.24)	6.69	4351

Rüdin analysed these two groups independently (Table 4). Consideration was given to other aspects such as alcoholism in the parents or mental illness in other relatives, in which case the families’ standard cards were re-sorted according to the question posed, and the proportions recalculated.

**Table 4:** Age distribution in category “both parents unaffected”.

Age of siblings at the time of ascertainment or at time of death	<14 years	14–68 years^1^	>68 Jahre^1^	Age unknown (not included)	Sum
**“Probands” -Patients with affective disorders**	-	530 (22^2^)	46 (0)	1	577 (22)
**“Secondary cases”—Affected siblings of probands**	-	53 (55^3^)	3 (3)	-	56 (58)
**“Other psychiatric conditions”—amongst the siblings of probands**	-	94 (97^4^)	4 (4)	-	98 (101)
**Unaffected siblings**	1222	1708 (1743)	111(111)	1	3042
**Sum**	1222	2385 (1917)	164 (118)	2	3773

^1^ The number of patients included in the calculation of morbid risk after applying the Weinberg proband and simple sib method are marked in brackets.

^2^ 11 families contained 2 probands. These were included as “secondary cases”, using the proband method.

^3^ Two families contained two probands and one secondary case, the latter was counted twice, using the proband method.

^4^ In three families there were two probands and one sibling with a different psychiatric condition. The latter was included twice, using the proband method.

The morbid risk was then calculated for various categories, and Rüdin found an increase of affective disorders in children of parents who had affective disorders. Contrary to the widely held assumption of a high inheritance rate of affective disorders [30), Rüdin calculated a substantially lower proportion with 7.43% affected children of healthy parents and 23.82% affected children of an affected parent. For an overview of Rudin’s study results see Table 5.

**Table 5:** Main problems and results of the study [28].

Problem	Results	Discussion
Are affective disorders hereditary diseases?	Prevalence of affective disorders amongst the descendants of affected patients: 30–35%.	Rüdin considered the inheritance of affective disorders to be proven.
Are affective disorders inherited in a simple Mendelian mode of inheritance?	Families with two healthy parents → 7.43% affected children. In a recessive mode of inheritance, 25% affected children are expected.	Recessive mendelian mode of inheritance had to be excluded.
	Families with one affected parent → 23.82% affected children. In a dominant mode of inheritance, 50% affected children are expected.	Dominant mendelian mode of inheritance had to be excluded.
Which mode of inheritance is the most likely for affective disorders?	Three-locus model with two recessive and one dominant factor, see Table 6.	Rüdin postulated this mode of inheritance to be the most likely for affective disorders.
Calculation of the morbid risk for a given proband		No sufficient results available to formulate a reliable heredity prognosis.
Is the occurence of affective disorders associated with dementia praecox?	Family history of dementia praecox in patients with affective disorders: 15.7%. Family history of dementia praecox in patients with dementia praecox: 45%.	Affective disorders and dementia praecox are inherited separately.
Sex ratio	Male: Female = 1:1.7	Sex ratio is influenced by higher suicide rates in male patients and more frequent hospitalisation in female patients.

Weinberg calculated the proportions expected from crossing different genetic strains, which were then compared with the morbid risks determined by Rüdin in order to find as close a match as possible. Because a simple recessive or dominant inheritance had to be excluded, Rüdin, with the aid of Weinberg’s calculations, took more and more complicated modes of Mendelian inheritance into consideration. Instead of looking more closely at other factors such as patients’ living conditions, comorbidity or other external influences, calculations with assumptions of up to 12 interacting alleles were made in order to come as close as possible to the expected proportions. With the assumption of a three-locus model with two recessive and one dominant factor, Rüdin’s proportions ultimately best matched those calculated by Weinberg, which is why he postulated this mode of inheritance as the most probable (Table 6). However, implementation of the “empirical heredity prognosis” was unsuccessful; it was still not possible to use a standardised formula to predict the probability that a particular individual would develop an illness. Hence the way one should conceive the genotype of a certain patient was still far from clear.

**Table 6:** Morbid risk of affective disorders in the offspring of various crosses. Comparison between empirical values and predicted values for a three-locus model with two recessive and one dominant factor.

	Predicted value from Mendelian crossing for three-locus model with two recessive and one dominant factor	Empirical morbid risk from Rüdin’s study
Cross unaffected x unaffected	10.50%	7.43%
Cross affected x unaffected	22.50%	23.82%
Cross unaffected x unaffected with otherwise positive family history	14.40%	14.94%
Cross half siblings	3.30%	1.42%
Cross affected x affected	77.40%	Not calculated

Rüdin emphasised the preliminarity of his results and called for further studies with larger sample size and avoidance of assortative mating. In order to better measure the effects of environmental factors, Rüdin initiated further family and twin studies into the inheritance of affective disorders at the GDA [41–43].

### Reception of Rüdin’s research and the further development of psychiatric genetics

Aubrey Lewis (1900–1975), in 1934, considered Rüdin’s studies the starting point in the field, refuting allegations against psychiatric genetics “that it was bad psychiatry and bad genetics” [44]. Lewis distinguished between Rüdin’s research and demands he made for changes in health policy, sharply criticising the eugenic measures in Germany’s “Law for the Prevention of Hereditarily Diseased Offspring”, albeit anonymously [45, 46]. In German-speaking psychiatry, there were well-known critics of Rüdin’s eugenic demands on society; important psychiatrists like Karl Jaspers (1883–1969), Oswald Bumke (1877–1950) and Eugen Bleuler (1857–1939) argued the mode of inheritance was unproven; external and social factors influenced disease course, and the right to personal self-determination had to be respected [15, 47–51] (for further insight into resistance of medical professionals against negative eugenic measures, see [52–54]).

Rüdin’s concept of an empirical heredity prognosis served as a methodological model for many subsequent studies at the DFA, known as the Munich School [55–60]. European and American scientists, some of whom had been fellows of the Genealogic-Demographic Department (GDA) of the DFA, used Rüdin’s research methodology in psychiatric genetic studies [27, 27, 60–65, 65–69]. The results of the twin and family studies undertaken at the Munich School remained valid in their methodology and results for decades [31, 42, 70–72].

### Publication bias

Whilst Rüdin actively published over decades [8], only one article reported the methodology he prized, namely his large-scale study on the inheritance of dementia praecox [24], with which he acquired his reputation as a psychiatric geneticist [15]. It is all the more surprising that the similarly laid out study on “manic-depressive insanity”, which was elaborately prepared and carried out over the course of years was never published [8, 28]. Contributing factors may include Rüdin, concentrating on his political career, paid less attention to the practical implementation of his research plans [15]. This is somewhat contradicted, by the fact that the study and manuscript were essentially completed. There is some evidence that Rüdin doubted his results. He concurred with eugenicist and heredity theorist Ludwig Plate (1862–1937), that diagnostic uncertainty necessitated skeptical application of Mendelian rules [73–75]. Most likely is that his demands for negative eugenic measures against patients with affective disorders and their families could not be justified on the grounds of the heredity figures he had calculated.

With the benefit of hindsight, the inheritance figures Rüdin calculated have been confirmed repeatedly [71, 76], and the search for replicable gene variants leading to the onset of affective disorders continues [77]. In a 1924 lecture Rüdin even recognised that environmental factors combined with disposition to illness, trigger onset of disease [78]. Rüdin’s actual results were therefore reasonably sound from today’s perspective, notwithstanding methodological limitations and that his studies were undertaken prior to knowledge of DNA, the double helix, and the intricacies of molecular genetics, epigenetics and endophenotypes [79–84].

Selective publication of positive results remains contentious today. The German human geneticist Peter Propping considers it the greatest danger to psychiatric genetics; “a silent coalition exists between an author and an editor: both are interested in publishing positive findings” [40]. He calls for a platform where all relevant results are accessible to the scientific public, to minimise bias in publishing [40]. In contrast to Propping, prominent scientists like Christiane Nüsslein-Volhard who received the Nobel Prize in Physiology or Medicine in 1995 recommend not publishing negative results, because science should increase knowledge, not merely produce more data. In a recent interview she points to methodological errors as a potential reason for negative results [85]. Rüdin too may have been dubious about his results and therefore refrained from publishing the study.

Rüdin had already clearly formulated his research aim before his study began. He unethically promulgated his eugenic ideology based on a selective and at times patently false reading of his results or even ignoring them [40, 86]. For further discussion on publication bias, see [87, 88].

### Rüdin’s involvement in National Socialist policy

In 1933, Rüdin chaired the committee for racial hygiene and racial policy at the ministry of the interior of the NS regime and collaborated on the “Law for the Prevention of Hereditarily Diseased Offspring” [8, 17]. For more detailed information about the role that scientists played in bolstering the racial theories of the NS regime see [3]. According to this law, all persons who had been determined to be hereditarily diseased according to medical science were to be compulsorily sterilised [17] (for the origins of the law see [8]). In his commentary on the law’s implementation, Rüdin justified sterilising psychiatric patients based on the results of his study of the inheritance of dementia praecox, extrapolating the allegedly proven inheritance to other psychiatric and neurologic diagnoses [15, 17, 89, 90]. In doing so, Rüdin reinterpreted his results as much more conclusive and reliable than he had in earlier commentaries, but without citing any later studies [24, 74].

As the story of the 1933 sterilisation-law shows, Rüdin’s research results were accepted uncritically as a scientific basis for legislation, strengthening the case for the National Socialists’ health and social policy. Between the law coming into force in 1934, until 1945, between 350,000 and 400,000 persons were sterilised [91–93]. The DFA, founded by Kraepelin and later led by Rüdin, was among the Institutes that issued registration forms required for sterilisation of patients [13]. The number of sterilisations only decreased when, in 1940 and 1941, the Nazis progressed to killing mentally ill and handicapped patients, in “*Aktion T4*”, under the euphemism “euthanasia” [94] (for the history of eugenics and euthanasia, see also [95–97]). Rüdin became aware of this secret operation at the end of 1939 at the latest, but was not directly involved in its preparation or execution [8]. However, his backing may have influenced important decisions at the highest political levels in favour of killing patients [98, 99]. Rüdin supported research projects which included killing designated patients for post mortem material [100, 101]. Also the meticulously recorded registration of patients in Rüdin’s studies later facilitated locating the victims for forced sterilisation and “*Aktion T4*” [98, 102]. Because his scientific interests were so consistent with Nazi ideology, the DFA was supported by the various centers of power in the National Socialist state [96, 103]. Rüdin did not consider his associations with these ideologies to compromise the scientific quality of his empirical studies [16]. In celebration of the tenth anniversary of the National Socialist state, Rüdin composed a laudation in praise of Adolf Hitler’s services to racial hygiene [104]. In a memorandum he referred to “euthanasia” as a component of “therapeutic reform” [10, 105].

Contemporary views of Rüdin’s work diverge widely, and are subject to ongoing controversy [13, 90, 99, 106]. Some would strip him entirely of his rank as a scientist [15]; others credit him with the establishment of modern psychiatric human genetics through the development of the “empirical heredity prognosis” [8, 31, 40]. Appeals to discontinue citing Rüdin’s scientific work have been put forward [107]. Certainly any scientific analysis of Rüdin’s works should provide information about his political role [108]. His legacy is deeply troubling but highly illustrative of the nexus between science, ideologies, ethics and humanity.
